# Клинические рекомендации «Заболевания и состояния, связанные с дефицитом йода»

**DOI:** 10.14341/probl12750

**Published:** 2021-04-16

**Authors:** Ф. М. Абдулхабирова, О. Б. Безлепкина, Д. Н. Бровин, Т. A. Вадина, Г. А. Мельниченко, Е. В. Нагаева, Л. В. Никанкина, В. А. Петеркова, Н. М. Платонова, А. А. Рыбакова, Т. В. Солдатова, Е. А. Трошина, Т. Ю. Ширяева

**Affiliations:** Национальный медицинский исследовательский центр эндокринологии; Национальный медицинский исследовательский центр эндокринологии; Национальный медицинский исследовательский центр эндокринологии; Национальный медицинский исследовательский центр эндокринологии; Национальный медицинский исследовательский центр эндокринологии; Национальный медицинский исследовательский центр эндокринологии; Национальный медицинский исследовательский центр эндокринологии; Национальный медицинский исследовательский центр эндокринологии; Национальный медицинский исследовательский центр эндокринологии; Национальный медицинский исследовательский центр эндокринологии; Национальный медицинский исследовательский центр эндокринологии; Национальный медицинский исследовательский центр эндокринологии; Национальный медицинский исследовательский центр эндокринологии

**Keywords:** клинические рекомендации, йодный дефицит, диффузный нетоксический зоб, узловой нетоксический зоб

## Abstract

Заболевания и состояния, связанные с дефицитом йода, — йододефицитные заболевания — широкий термин, включающий в себя не только нарушение структуры щитовидной железы, но и состояния, связанные с дефицитом тиреоидных гормонов.

Клинические рекомендации — это основной рабочий инструмент практикующего врача, как специалиста, так и врача узкой практики. Лаконичность, структурированность сведений об определенной нозологии, методов ее диагностики и лечения, базирующихся на принципах доказательной медицины, позволяют в короткий срок дать тот или иной ответ на интересующий вопрос специалисту, добиваться максимальной эффективности и персонализации лечения.

Данные клинические рекомендации включают в себя алгоритмы диагностики и лечения диффузного нетоксического зоба и узлового/многоузлового зоба у взрослых и детей. Кроме того, настоящие клинические рекомендации содержат информацию о способах адекватной эпидемиологической оценки йододефицитных заболеваний с помощью таких маркеров, как процентное соотношение зоба у школьников, медианная концентрация йода в моче, уровень неонатального тиреотропного гормона, медиана тиреоглобулина у детей и взрослых, а также об основных методах и группах эпидемиологических исследований йододефицитных заболеваний.

## Abstract (en)

Iodine deficiency disorders is a sweeping term that includes structural and functional impairment of the thyroid gland.

These clinical guidelines include algorithms for the diagnosis and treatment of euthyroid goiter and nodular/ multinodular goiter in adults and children. In addition, these clinical guidelines contain information on methods for an adequate epidemiological assessment of iodine deficiency disorders using such markers as the percentage of goiter in schoolchildren, the median urinary iodine concentration, the level of neonatal TSH, the median thyroglobulin in children and adults. As well from these clinical guidelines, you can get to know the main methods and groups of epidemiological studies of iodine deficiency disorders.

## СПИСОК СОКРАЩЕНИЙ

 

## ТЕРМИНЫ И ОПРЕДЕЛЕНИЯ

Йододефицитные заболевания — патологические состояния, обусловленные дефицитом йода, которые могут быть предотвращены посредством обеспечения населения необходимым количеством йода.

Нетоксический зоб — заболевание, характеризующееся диффузным или узловым увеличением щитовидной железы (ЩЖ) без нарушения ее функции.

Диффузный нетоксический (эутиреоидный) зоб — увеличение ЩЖ без нарушения ее функции, определяемое пальпаторно или методом ультразвукового исследования (УЗИ).

Спорадический зоб — диффузное увеличение ЩЖ, обусловленное, как правило, врожденными (генетическими) или приобретенными дефектами синтеза гормонов ЩЖ.

Эндемический зоб — увеличение ЩЖ, обусловленное дефицитом йода, у части населения, проживающего в определенном регионе.

Узловой или многоузловой зоб — собирательное клиническое понятие, объединяющее все пальпируемые очаговые образования в ЩЖ, которые имеют различные морфологические характеристики.

Узловой или многоузловой коллоидный зоб — заболевание ЩЖ, возникающее в результате очаговой пролиферации тиреоцитов и накопления коллоида.

Узловой или многоузловой токсический зоб — состояние, при котором стойкая патологическая гиперпродукция тиреоидных гормонов обусловлена формированием в ЩЖ автономно функционирующих тиреоцитов.

Функциональная автономия ЩЖ — независимый от влияния тиреотропного гормона гипофиза захват йода и продукция тироксина тиреоцитами.

Дефицит йода — потребление йода ниже рекомендованной суточной потребности организма в мкг для каждой возрастной группы (90 мкг у детей и 150 мкг у взрослых).

Йодированная соль — поваренная соль, содержащая фиксированное количество солей йода (йодат калия), использующаяся для массовой профилактики йододефицитных заболеваний.

Кретинизм — крайняя степень задержки умственного и физического развития, связанная с недостатком тиреоидных гормонов во внутриутробном периоде.

Кластерный анализ — математическая процедура, позволяющая на основе схожести количественных значений нескольких признаков, свойственных каждому объекту (например, испытуемого) какого-либо множества, сгруппировать эти объекты в определенные классы, или кластеры. Главное назначение кластерного анализа — разбиение множества исследуемых объектов и признаков на однородные в соответствующем понимании группы или кластера.

Неонатальный скрининг на гипотиреоз — система раннего выявления недостаточности ЩЖ у новорожденных.

Тиреотоксикоз — клинический синдром, обусловленный длительным избытком гормонов ЩЖ в организме и их токсическим действием на различные органы и ткани. Синдром тиреотоксикоза развивается как при заболеваниях ЩЖ, так и при заболеваниях других органов и патологических состояниях.

 

1.1. Определение заболевания или состояния (группы заболеваний или состояний)

Йододефицитные заболевания (ЙДЗ) — термин, объединяющий состояния и нарушения, вызванные йодным дефицитом (ВОЗ, 2007 г.).

ЙДЗ объединяют не только патологию ЩЖ, развившуюся вследствие дефицита йода, но и патологические состояния, обусловленные дефицитом тиреоидных гормонов. Спектр ЙДЗ представлен в таблице 1.

**Table:** Таблица 1. Спектр йододефицитной патологии (ВОЗ, 2001 г.)

Внутриутробный период	Аборты.Мертворождение.Врожденные аномалии.Повышение перинатальной смертности.Повышение детской смертности.Неврологический кретинизм (умственная отсталость, глухонемота, косоглазие).Микседематозный кретинизм (умственная отсталость, гипотиреоз, карликовость).Психомоторные нарушения
Новорожденные	Неонатальный гипотиреоз
Дети и подростки	Нарушения умственного и физического развития
Взрослые	Зоб и его осложнения.Йодиндуцированный тиреотоксикоз
Все возрасты	Зоб.Гипотиреоз.Нарушения когнитивной функции.Повышение поглощения радиоактивного йода при ядерных катастрофах

1.2. Этиология и патогенез заболевания или состояния (группы заболеваний или состояний)

I является обязательным структурным компонентом гормонов ЩЖ, которые, в свою очередь, обеспечивают полноценное развитие и функционирование человеческого организма. Основными природными источниками I для человека являются продукты растительного и животного происхождения, питьевая вода, воздух. Суточная потребность в данном элементе составляет:

Недостаток I в почве приводит к снижению содержания этого микроэлемента в продуктах питания, производимых в этой местности, а потребляющие их люди страдают от йододефицита [1][2]. Дефицит I обладает многочисленными негативными последствиями в отношении развития и формирования организма человека. Известно, что наибольшую опасность представляет недостаточное поступление I в организм на этапе внутриутробного развития и в раннем детском возрасте. Изменения, вызванные йододефицитом (ЙД) в эти периоды жизни, проявляются необратимыми дефектами в интеллектуальном и физическом развитии детей. В таблице 2 отражена роль I как субстрата для выработки тиреоидных гормонов, играющих ключевую роль в формировании центральной нервной системы [3][4].

**Table:** Таблица 2. Роль тиреоидных гормонов в формировании центральной нервной системы

Этап	Срок гестации	ЦНС	Тиреоидные гормоны
I	До 12–15 нед	Закладка основных структур головного мозга	Матери
II	15–40 нед	Формирование ЦНС, созревание нейронов, миелинизация, синаптогенез	Матери и плода
III	Постнатальный период	Миелинизация ЦНС, формирование мозжечка, зубчатого гиппокампа	Новорожденного

Однако весь спектр йододефицитной патологии широк и простирается от репродуктивных нарушений до специфических заболеваний ЩЖ, включая функциональную автономию и йодиндуцированный тиреотоксикоз как одно из самых тяжелых проявлений ЙДЗ в регионах с различным уровнем ЙД в питании [5].

На ранних стадиях развития зоба (у детей, подростков и молодых людей) происходит компенсаторная гипертрофия тиреоцитов. Несомненно, что все реакции адаптации стимулируются и контролируются тиреотропным гормоном (ТТГ). Однако, как было показано во многих работах, уровень ТТГ при диффузном нетоксическом зобе (ДНЗ) не повышается. В ходе ряда исследований in vivo и in vitro были получены новые данные об ауторегуляции ЩЖ I и аутокринными ростовыми факторами. По современным представлениям, повышение продукции ТТГ или повышение чувствительности к нему тиреоцитов имеет лишь второстепенное значение в патогенезе йододефицитного зоба. Основная роль при этом отводится аутокринным ростовым факторам, таким как инсулиноподобный ростовой фактор 1-го типа, эпидермальный ростовой фактор и фактор роста фибробластов, которые в условиях снижения содержания I в ЩЖ оказывают мощное стимулирующее воздействие на тиреоциты [6]. Экспериментально было показано, что при добавлении в культуру тиреоцитов калия йодида** наблюдалось снижение ТТГ-индуцируемого цАМФ (циклического аденозинмонофосфата), опосредованное экспрессией мРНК инсулиноподобным ростовым фактором 1-го типа, с полным ее прекращением при значительном увеличении дозы калия йодида**. Хорошо известно, что I сам по себе не только является субстратом для синтеза тиреоидных гормонов, но и регулирует рост и функцию ЩЖ. Пролиферация тиреоцитов находится в обратной зависимости от интратиреоидного содержания I. Высокие дозы I ингибируют его поглощение, его органификацию, синтез и секрецию тиреоидных гормонов, поглощение глюкозы и аминокислот. I, поступая в тиреоцит, вступает во взаимодействие не только с тирозильными остатками в тиреоглобулине, но и с липидами. Образованные в результате этого соединения (йодолактоны и йодальдегиды) служат основными физиологическими блокаторами продукции аутокринных ростовых факторов. В ЩЖ человека идентифицировано много различных йодолактонов, которые образуются за счет взаимодействия мембранных полиненасыщенных жирных кислот (арахидоновой, докозагексаеновой и др.) с I в присутствии лактопероксидазы и перекиси водорода. В условиях хронической йодной недостаточности возникает снижение образования йодлипидов — веществ, сдерживающих пролиферативные эффекты аутокринных ростовых факторов (инсулиноподобного ростового фактора 1-го типа, фактора роста фибробластов, эпидермального ростового фактора) [7]. Кроме того, при недостаточном содержании I происходит повышение чувствительности этих аутокринных ростовых факторов к ростовым эффектам ТТГ, снижается продукция трансформирующего фактора роста-b, который в норме служит ингибитором пролиферации, активируется ангиогенез. Все это приводит к увеличению ЩЖ, образованию йододефицитного зоба. В целом развитие ДНЗ может зависеть и от многих других факторов, которые до конца не изучены. Помимо йодного дефицита, к другим причинам, имеющим отношение к развитию зоба, относят курение, прием некоторых лекарственных средств, экологические факторы. Имеют значение также пол, возраст, наследственная предрасположенность. При эндемическом зобе генетическая предрасположенность может реализоваться только при наличии соответствующего внешнего фактора — ЙД в окружающей среде [8].

1.3. Эпидемиология заболевания или состояния (группы заболеваний или состояний)

По данным ВОЗ, в условиях ЙД живут более 2 млрд человек, среди них почти у 700 млн человек выявлен эндемический зоб. В Российской Федерации не существует территорий, на которых население не подвергалось бы риску развития ЙДЗ. Считается, что район свободен от ЙД, если средняя концентрация I в моче у населения превышает 100 мкг/л. Среднее потребление I населением РФ намного ниже рекомендуемого и составляет 40–80 мкг в сутки. В эндемичных районах частота зоба у детей допубертатного возраста превышает 5% [9]. Распространенность диффузного эндемического зоба в различных регионах России варьирует от 5,2 до 70% и в среднем по стране составляет 31% [10][11][12][13][14]. ДНЗ преимущественно встречается у детей, подростков и лиц молодого возраста. Более чем в 50% случаев он развивается до 20-летнего возраста, причем у женщин зоб развивается в 2–3 раза чаще, чем у мужчин. Как правило, риск развития ДНЗ многократно возрастает в те периоды, когда повышенная потребность в I (детский возраст, пубертатный период, беременность, кормление грудью) не восполняется адекватно [15]. Применение йодированной соли во многих случаях способно ликвидировать йодный дефицит [16]. Для оценки степени тяжести ЙД и успеха профилактических программ необходимо проведение четко спланированных репрезентативных популяционных исследований. Критерии оценки тяжести ЙД и методы эпидемиологических исследований представлены в дополнительной информации.

1.4. Особенности кодирования заболевания или состояния (группы заболеваний или состояний) по Международной статистической классификации болезней и проблем, связанных со здоровьем

E01.0 Диффузный (эндемический) зоб, связанный с йодной недостаточностью.

E01.1 Многоузловой (эндемический) зоб, связанный с йодной недостаточностью.

E01.2 Зоб (эндемический), связанный с йодной недостаточностью, неуточненный.

E01.8 Другие болезни щитовидной железы, связанные с йодной недостаточностью, и сходные состояния.

E02 Субклинический гипотиреоз вследствие йодной недостаточности.

E04.0 Нетоксический диффузный зоб.

E04.1 Нетоксический одноузловой зоб.

E04.2 Нетоксический многоузловой зоб.

E04.8 Другие уточненные формы нетоксического зоба.

E04.9 Нетоксический зоб неуточненный.

E07.9 Болезнь щитовидной железы неуточненная.

1.5. Классификация заболевания или состояния (группы заболеваний или состояний)

ЙД в питании приводит к развитию следующих заболеваний ЩЖ:

1.6. Клиническая картина заболевания или состояния (группы заболеваний или состояний)

Клиническая симптоматика может либо отсутствовать, либо проявляться косметическим дефектом или синдромом сдавления трахеи, пищевода, что зависит от степени увеличения объема ЩЖ. При загрудинном зобе больших размеров может отмечаться деформация шеи, а иногда, за счет компрессионного синдрома, набухание шейных вен. Пальпаторно могут определяться узловые образования, иногда слегка болезненные за счет перерастяжения капсулы ЩЖ.

 

Критерии установления диагноза диффузного зоба: на основании патогномоничных данных:

1. анамнестических данных;

2. физикального обследования;

3. лабораторных исследований;

4. инструментального обследования.

Критерии установления диагноза узлового/многоузлового зоба: на основании патогномоничных данных:

1. анамнестических данных;

2. физикального обследования;

3. лабораторных исследований;

4. инструментального обследования.

2.1. Жалобы и анамнез

2.1.1. Диффузный зоб

Нетоксический зоб небольших размеров обычно протекает бессимптомно. Как правило, зоб является случайной находкой. В подавляющем большинстве случаев в условиях легкого и умеренного ЙД небольшое увеличение ЩЖ обнаруживают лишь при целенаправленном обследовании. В условиях тяжелого ЙД зоб может достигать гигантских размеров. При сборе анамнеза рекомендуется оценивать местные признаки (изменение голоса, дисфагия и др.), клинические признаки нарушения функции ЩЖ, медицинский анамнез вмешательств на ЩЖ, семейный анамнез, включая наличие УЗ и медуллярного рака у родственников, предшествующее облучение области головы и шеи, проживание в условиях ЙД. На фоне ДНЗ в дальнейшем также может развиться УЗ и сформироваться функциональная автономия ЩЖ, которая служит одной из основных причин развития тиреотоксикоза в йододефицитных регионах.

2.1.2. Узловой/многоузловой зоб

Нетоксический зоб небольших размеров обычно протекает бессимптомно. Как правило, зоб является случайной находкой. В подавляющем большинстве случаев в условиях легкого и умеренного йодного дефицита небольшое увеличение ЩЖ обнаруживают лишь при целенаправленном обследовании. При сборе анамнеза рекомендуется оценивать местные признаки (изменение голоса, дисфагия и др.), признаки нарушения функции ЩЖ, медицинский анамнез вмешательств на ЩЖ, семейный анамнез, включая наличие узлового зоба и медуллярного рака у родственников, предшествующее облучение области головы и шеи, проживание в условиях йодного дефицита.

2.2. Физикальное обследование

2.2.1. Диффузный зоб

Для оценки степени увеличения ЩЖ методом пальпации ВОЗ (2001) рекомендована следующая классификация [17]:

0-я (нулевая) степень — зоба нет (объем каждой доли не превышает объем дистальной фаланги большого пальца руки обследуемого);

1-я степень — зоб пальпируется, но не виден при нормальном положении шеи. Сюда же относятся узловые образования, не приводящие к увеличению самой железы;

2-я степень — зоб четко виден при нормальном положении шеи.

Важно отметить, что не всегда определяемые пальпаторно размеры ЩЖ совпадают с истинными, например, по причинам анатомических особенностей строения шеи, низкого расположения самой ЩЖ или загрудинного зоба. Если по результатам пальпации сделан вывод об увеличении размеров ЩЖ или о наличии узловых образований, пациенту показано проведение УЗИ ЩЖ.

Уровень убедительности рекомендаций C. Уровень достоверности доказательств 5.

2.2.2. Узловой/многоузловой зоб

При пальпации может определяться увеличение ЩЖ. Классификация размеров зоба (ВОЗ, 2001) представлена выше. Если по результатам пальпации сделан вывод об увеличении размеров ЩЖ или о наличии узловых образований, пациенту показано проведение УЗИ ЩЖ.

Уровень убедительности рекомендаций C. Уровень достоверности доказательств 5.

2.3. Лабораторные диагностические исследования

2.3.1. Диффузный зоб

Уровень убедительности рекомендаций C. Уровень достоверности доказательств 5.

Уровень убедительности рекомендаций C. Уровень достоверности доказательств 5.

Уровень убедительности рекомендаций C. Уровень достоверности доказательств 5.

Комментарии. Гипотиреоз вследствие йодной недостаточности характерен для районов с тяжелым ЙД (потребление менее 20 мкг/сут). В районах с легким и умеренным ЙД гипотиреоз по причине йодного дефицита не встречается.

2.3.2. Узловой/многоузловой зоб

Уровень убедительности рекомендаций C. Уровень достоверности доказательств 5.

Уровень убедительности рекомендаций C. Уровень достоверности доказательств 5.

Уровень убедительности рекомендаций A. Уровень достоверности доказательств 2.

Уровень убедительности рекомендаций C. Уровень достоверности доказательств 4.

2.4. Инструментальные диагностические исследования

2.4.1. Диффузный зоб

V щж = [(Ш пр × Д пр × Т пр ) + (Ш л × Д л × Т л )] × 0,479.

Уровень убедительности рекомендаций А. Уровень достоверности доказательств 2.

Комментарии. У взрослых диффузный зоб диагностируют, если объем железы по данным УЗИ превышает 18 мл у женщин и 25 мл у мужчин.

Для оценки зоба у детей используются, как правило, данные пальпации или нормативы, принятые для эпидемиологических исследований, где объем ЩЖ сопоставляется с площадью поверхности тела ребенка (см. табл. 8).

2.4.2. Узловой/многоузловой зоб

V щж = [(Ш пр × Д пр × Т пр ) + (Ш л × Д л × Т л )] × 0,479.

Уровень убедительности рекомендаций А. Уровень достоверности доказательств 2.

Уровень убедительности рекомендаций А. Уровень достоверности доказательств 2.

Уровень убедительности рекомендаций А. Уровень достоверности доказательств 1.

Комментарии. Классификация EU-TIRADS используется для того, чтобы определить дальнейшую тактику ведения пациентов, у которых выявили узловые изменения в щитовидной железе.

Классификация EU-TIRADS:

Комментарии. Изначально классификация EU TIRADS была разработана для взрослых, однако, учитывая схожие ультразвуковые признаки, может использоваться и у детей.

Уровень убедительности рекомендаций B. Уровень достоверности доказательств 2.

Уровень убедительности рекомендаций C. Уровень достоверности доказательств 3.

Уровень убедительности рекомендаций C. Уровень достоверности доказательств 4.

Уровень убедительности рекомендаций C. Уровень достоверности доказательств 5.

Комментарии. Заключения, содержащие только описательную часть, а также заключения без конкретного цитологического диагноза («атипичных клеток не обнаружено», «данных за рак нет» и т.п.) расцениваются как неинформативные. В этих ситуациях необходимо проконсультировать готовые цитологические препараты у другого независимого морфолога или повторить ТАБ в специализированном лечебном учреждении.

Комментарии. Заключение цитологического исследования должно включать одну из диагностических категорий, которое позволит клиницисту поставить клинический диагноз и определить оптимальную лечебную тактику в отношении каждого конкретного больного.

Комментарии. В детском возрасте классификация Бетесда также удобна для использования. Однако более высокий риск выявления рака ЩЖ при наличии узлового образования меняет отношение к категории «доброкачественные изменения» и «фолликулярная неоплазия» (табл. 4) [43].

**Table:** Таблица 3. Рекомендуемые диагностические категории и рекомендации по тактике ведения по классификации Бетесда (2017).

Диагностические категории	Тактика ведения
I — неинформативный пунктат	Повторная ТАБ
II — доброкачественные изменения	Наблюдение
III — атипия неопределенного значения (или изменения фолликулярного эпителия неясного значения)	Повторная ТАБ/молекулярно-генетическое исследование/гемитиреоидэктомия
IV — фолликулярная неоплазия или подозрение на фолликулярную неоплазию	Молекулярно-генетическое исследование/ гемитиреоидэктомия
V — подозрение на злокачественную опухоль	Гемитиреоидэктомия или тиреоидэктомия
VI — злокачественная опухоль	Гемитиреоидэктомия или тиреоидэктомия

**Table:** Таблица 4. Сравнение риска малигнизации по классификации Бетесда у взрослых и детей по категориям II и IV

Диагностические категории	Онкологический риск,взрослые, %	Онкологический риск, дети, %
II — доброкачественные изменения	0–3	8
IV — фолликулярная неоплазия или подозрение на фолликулярную неоплазию	15–30	30

Уровень убедительности рекомендаций C. Уровень достоверности доказательств 5.

Уровень убедительности рекомендаций C. Уровень достоверности доказательств 4.

2.5. Иные диагностические исследования

2.5.1. Диффузный зоб

Уровень убедительности рекомендаций C. Уровень достоверности доказательств 5.

2.5.2. Узловой/многоузловой зоб

Уровень убедительности рекомендаций C. Уровень достоверности доказательств 5.

Комментарии. Наиболее часто для сцинтиграфии ЩЖ используется натрия пертехнетат [99mTc], йобенгуан [I123], реже натрия йодид [I131]. Натрия пертехнетат [99mTc] имеет короткий период полураспада (6 ч), что значительно уменьшает дозу облучения. При функциональной автономии изотоп накапливает активно функционирующий узел, при этом окружающая тиреоидная ткань находится в состоянии супрессии. В ряде случаев автономия может носить диффузный характер за счет диссеминации автономно функционирующих участков по всей ЩЖ.

Уровень убедительности рекомендаций C. Уровень достоверности доказательств 4.

Уровень убедительности рекомендаций C. Уровень достоверности доказательств 5.

 

3.1. Диффузный зоб

3.1.1. Консервативное лечение

Уровень убедительности рекомендаций C. Уровень достоверности доказательств 5.

Комментарии. Целью лечения ДНЗ является нормализация или уменьшение объема ЩЖ.

На сегодняшний день существует три варианта консервативной терапии ДНЗ:

Основными преимуществами монотерапии калия йодидом** являются ее этиотропный характер (йододефицитный зоб — практически единственное заболевание в эндокринологии, при котором осуществима этиотропная терапия), безопасность, отсутствие необходимости в подборе дозы и в проведении частых гормональных исследований.

Терапия левотироксином натрия** или комбинированная терапия являются предпочтительными при большом объеме ЩЖ или отсутствии эффекта от монотерапии калия йодидом**. Доза препарата должна быть такой, чтобы уровень ТТГ был снижен до нижней границы нормальных значений. Однако при выборе такой тактики терапии существует риск развития медикаментозного тиреотоксикоза, необходимость подбора дозы, что требует частых исследований.

Длительность терапии 6–12 мес, далее при достижении цели лечения обязательно использование йодированной соли в питании.

Уровень убедительности рекомендаций C. Уровень достоверности доказательств 3.

Комментарии. Применение препаратов калия йодида** в данной возрастной группе не показано с учетом возможного риска индукции развития и декомпенсации функциональной автономии ЩЖ.

Уровень убедительности рекомендаций C. Уровень достоверности доказательств 5.

Комментарии. При этом следует помнить, что даже при достаточном потреблении I в период беременности объем ЩЖ закономерно несколько увеличивается.

Уровень убедительности рекомендаций В. Уровень достоверности доказательств 2.

3.1.2. Хирургическое лечение

Уровень убедительности рекомендаций C. Уровень достоверности доказательств 4.

3.1.3. Иное лечение

**Figure:**
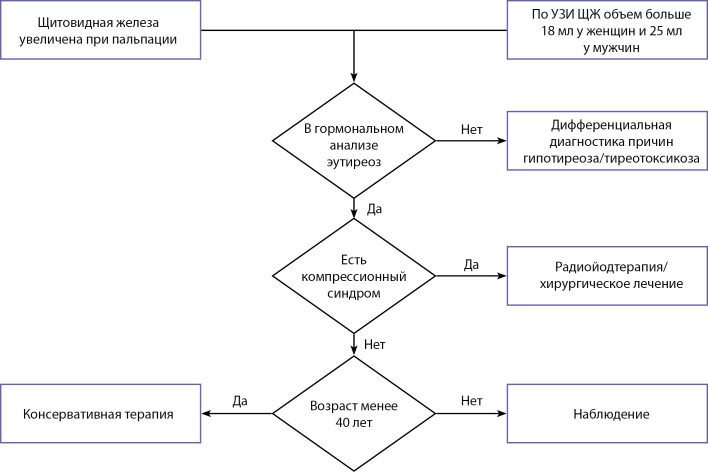
Рисунок 1. Схема ведения пациентов при диффузном зобе.

Уровень убедительности рекомендаций A. Уровень достоверности доказательств 1.

3.2. Узловой/многоузловой зоб

3.2.1. Консервативное лечение

Методов консервативного лечения узлового нетоксического зоба не существует.

Уровень убедительности рекомендаций C. Уровень достоверности доказательств 5.

3.2.2. Хирургическое лечение

Уровень убедительности рекомендаций C. Уровень достоверности доказательств 5.

Комментарии. Если в качестве метода лечения УТЗ/МТЗ выбрана операция, у пациентов с манифестным тиреотоксикозом необходимо достижение эутиреоза на фоне терапии антитиреоидными препаратами. Операцией выбора при МТЗ является тиреоидэктомия. Операцией выбора при УТЗ является гемитиреоидэктомия пораженной доли ЩЖ.

После неадекватных по объему операции по поводу УТЗ/МТЗ методом выбора лечения тиреотоксикоза является радиойодтерапия.

3.2.3. Иные виды лечения

Уровень убедительности рекомендаций A. Уровень достоверности доказательств 2.

Комментарии. Длительное консервативное лечение антитиреоидными препаратами целесообразно лишь в случаях невозможности выполнить радикальное лечение (пожилой возраст, наличие тяжелой сопутствующей патологии). Предварительное лечение антитиреоидными препаратами перед проведением радиойодтерапии при УТЗ/МТЗ должно обсуждаться для пациентов, имеющих повышенный риск развития осложнений в связи с усилением тиреотоксикоза, включая пожилых пациентов и тех, у кого имеются заболевания сердечно-сосудистой системы или тяжелый тиреотоксикоз. Для лечения УТЗ/МТЗ применяют достаточно высокие дозы йобенгуана [I123] (350–450 Гр), поскольку он поглощается только автономными участками и частота развития гипотиреоза значительно ниже, чем при терапии ДТЗ. Целью лечения является деструкция автономно функционирующей ткани с восстановлением эутиреоза. Наблюдение пациентов после радиойодтерапии подразумевает определение СТ4 и ТТГ 1 раз в 1–2 мес. Если тиреотоксикоз сохраняется в течение 6 мес после лечения, рекомендовано повторное выполнение радиойодтерапии.

Уровень убедительности рекомендаций C. Уровень достоверности доказательств 4.

Комментарии. Резекция может быть отсрочена при доброкачественных результатах ТАБ и отсутствии тиреотоксикоза.

Комментарии. Проведение радиойодтерапии доброкачественного по ТАБ УТЗ (Бетесда II) возможно при высоком риске хирургического вмешательства, отказе родителей и ребенка от оперативного вмешательства (достигшего 15 лет при получении информированного согласия, при завершении пубертатного периода: замедлении роста, снижении митотической активности и основного обмена).

**Figure:**
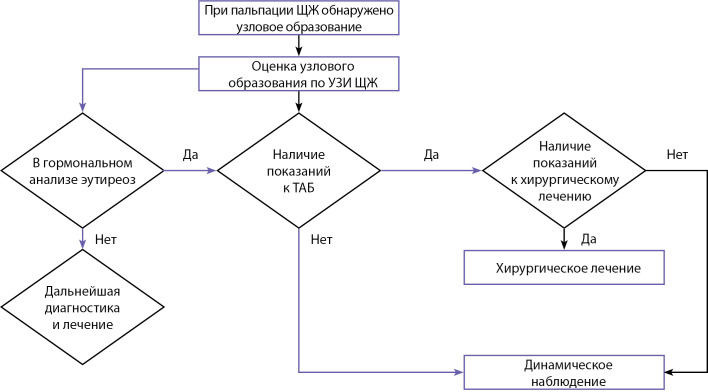
Рисунок 2. Схема ведения пациентов при узловом/многоузловом зобе.

Уровень убедительности рекомендаций C. Уровень достоверности доказательств 4.

Комментарии. Не показаны чрескожные инъекции этанола**:

 

Специфических реабилитационных мероприятий в отношении данных пациентов не разработано.

 

Уровень убедительности рекомендаций C. Уровень достоверности доказательств 5.

Уровень убедительности рекомендаций C. Уровень достоверности доказательств 5.

Комментарии. Профилактика в масштабе определенных групп повышенного риска по развитию ЙДЗ осуществляется путем приема фармакологических средств, содержащих физиологическую дозу калия йодида**.

Комментарии. Всеобщее йодирование соли рекомендовано ВОЗ в качестве универсального, высокоэффективного метода массовой йодной профилактики. Всеобщее йодирование соли означает, что практически вся соль для употребления человеком (т.е. продающаяся в магазинах и используемая в пищевой промышленности) должна быть йодирована. Для достижения оптимального потребления I (150 мкг/сут для взрослых) ВОЗ и Международный совет по контролю за ЙДЗ рекомендуют добавление в среднем 20–40 мг I на 1 кг соли. В РФ постановлением главного санитарного врача рекомендовано добавление в среднем 40±15 мг I на кг соли. В качестве йодирующей добавки рекомендовано использовать йодат калия. Применение йодированной соли во многих случаях способно ликвидировать йодный дефицит.

 

Госпитализация плановая. Помощь стационарная.

Показания для плановой госпитализации в медицинскую организацию (стационар):

1. подготовка к проведению оперативного вмешательства.

Показания к выписке пациента из медицинской организации:

1. стойкое улучшение состояния, когда пациент может без ущерба для здоровья продолжить лечение в амбулаторно-поликлиническом учреждении или домашних условиях.

 

7.1. Эпидемиологическая оценка йододефицитных заболеваний

7.1.1. Исследование медианной концентрации I в моче

В настоящее время экскреция йода с мочой рассматривается как основной эпидемиологический показатель, характеризующий йодную обеспеченность того или иного региона. Этот показатель является высокочувствительным, быстро реагирует на изменения в потреблении йода и поэтому имеет важнейшее значение не только для оценки эпидемиологической ситуации, но и для осуществления контроля программ профилактики йододефицитных заболеваний.

С мочой выводится 80–90% потребляемого с пищей йода. Концентрация йода в разовой порции мочи хорошо коррелирует с уровнем йода в суточной моче и отражает поступление йода в организм непосредственно на момент исследования. Так как уровень йода в моче у конкретного лица меняется не только ежедневно, но и в течение дня, данные определения йода можно использовать только для оценки йодной обеспеченности популяции в целом. Этот метод пригоден только для эпидемиологических исследований. В связи с очень неравномерным распределением уровня йода в образцах мочи предпочтительнее оценивать медиану, а не среднее значение.

Критерии оценки потребления йода населением, основанные на медианной концентрации йода в моче, представлены в таблице 5.

**Table:** Таблица 5. Критерии оценки потребления йода населением, основанные на медианной концентрации йода в моче у детей школьного возраста

Медианная концентрация йода в моче, мкг/сут	Потребление йода	Эпидемиологическая ситуация в регионе
<20	Недостаточное	Тяжелый йодный дефицит
20–49	Недостаточное	Йодный дефицит средней тяжести
50–99	Недостаточное	Йодный дефицит легкой степени
100–199	Адекватное	Нормальная йодная обеспеченность
200–299	Превышает норму	Риск развития йодиндуцированного тиреотоксикоза
>300	Избыточное	Риск развития неблагоприятных последствий для здоровья (йодиндуцированный гипотиреоз, аутоиммунные заболевания щитовидной железы)

Выбор репрезентативной группы для оценки потребления йода в популяции обеспечивается путем проведения кластерного исследования. Наиболее эффективным и обоснованным с практической точки зрения является проведение исследования на базе школ. При планировании и подготовке работы на основании списка всех школ данного региона тем или иным методом определяются 30 кластеров. В каждом кластере проводится исследование не менее 30 образцов мочи. Для стран с большим населением или имеющих на своей территории несколько различных экологических зон проводится несколько независимых исследований. Проведение репрезентативного 30-кластерного исследования является дорогостоящим и трудоемким, поэтому для мониторинга программ профилактики ЙДЗ достаточно обследовать контрольные районы, в которых исходно наблюдался тяжелый или средней тяжести йодный дефицит. В каждом из контрольных районов методом рандомизации определяются не менее 3 школ. В каждой из выбранных школ исследуется не менее 30 образцов мочи и соли, используемой в семьях учеников. На фоне проведения профилактических мероприятий такие исследования в контрольных районах проводятся один раз в 2 года.

В таблицах 6 и 7 суммированы основные методы эпидемиологических исследований и группы населения, наиболее приемлемые для проведения таких исследований.

**Table:** Таблица 6. Основные методы эпидемиологических исследований

(Основной принцип — «30 кластеров + не менее 30 образцов мочи из каждого кластера»)1.Кластерные, пропорциональные количеству населения исследования:-исследования на базе школ;-«подворовые» обходы.2.Альтернативные методы (например, первичное обследование в школах регионов, не имеющих никаких данных о ситуации с ЙДЗ; для географически неоднородных регионов и т.д.)

**Table:** Таблица 7. Основные группы для эпидемиологических исследований

Дети школьного возраста (8–10 лет) — основная группа.Новорожденные (при условии уже проведенного первичного скринингового исследования йодной обеспеченности в регионе)

7.2. Определение частоты зоба в популяции

Изменения объема ЩЖ, как правило, связаны с уровнем поступления йода в организм, однако изменение объема железы в ответ на изменившееся потребление йода происходит в течение нескольких месяцев или даже нескольких лет. На степень увеличения ЩЖ оказывают влияние степень йодного дефицита, длительность проживания в условиях нехватки йода, профилактические мероприятия, пол, возраст и т.д.

Таким образом, в настоящее время распространенность зоба как критерий оценки йодной обеспеченности практически перестал учитываться. Это связано в первую очередь с тем, что основной акцент смещен на контроль за выполнением программы всеобщего йодирования соли. Распространенность зоба является косвенным показателем уровня потребления йода и выраженности йодного дефицита и меняется спустя достаточно длительный срок после нормализации потребления йода. Кроме того, в настоящее время отсутствуют общепринятые нормативы рассчитываемого при помощи УЗИ объема ЩЖ у детей. Определение частоты зоба в популяции имеет определенное значение для оценки степени тяжести йодного дефицита, которое проводится до начала профилактических мероприятий. В этом случае наиболее целесообразным является определение частоты зоба у детей 8–10 лет. У детей младше 8 лет определение объема ЩЖ представляет определенные технические сложности, а у детей более старшего возраста увеличение объема ЩЖ может быть обусловлено началом пубертатного периода. Для оценки степени увеличения ЩЖ методом пальпации рекомендована классификация ВОЗ (см. пункт 2.2). Чувствительность и специфичность метода пальпации для оценки степени зоба довольно низкие. Поэтому для точного определения размеров и объема ЩЖ в рамках эпидемиологического исследования рекомендуется проведение УЗИ с подсчетом объема ЩЖ (см. пункт 2.4). У ребенка объем ЩЖ зависит от степени физического развития, поэтому перед исследованием измеряются рост и вес ребенка и по специальной шкале или по формуле вычисляется площадь поверхности тела. У детей объем ЩЖ сопоставляется с нормативными показателями (в зависимости от площади поверхности тела, табл. 8).

**Table:** Таблица 8. Нормативные показатели объема щитовидной железы у детей для эпидемиологических исследований (верхний предел нормальных значений — 97 перцентиль) (ВОЗ, 1997) [89]

Площадьповерхности тела, м2	0,8	0,9	1,0	1,1	1,2	1,3	1,4	1,5	1,6	1,7
Мальчики	4,7	5,3	6,0	7,0	8,0	9,3	10,7	12,2	14,0	15,8
Девочки	4,8	5,9	7,1	8,3	9,5	10,7	11,9	13,1	14,3	15,6

Представленные в таблице показатели верхних пределов нормальных значений объема ЩЖ базируются на результатах обследования детей, проживающих в йодообеспеченных регионах. Общепринятых стандартов для объема ЩЖ у детей в настоящее время не существует, что вызывает определенные разногласия при трактовке результатов. Принятые нормативы объема щитовидной железы у детей ВОЗ рекомендует использовать только при проведении эпидемиологических исследований. В клинической практике данные нормативы не применяются, оценка зоба у детей в практическом здравоохранении проводится методом пальпации.

7.3. Другие показатели, используемые для оценки йодной обеспеченности

В литературе можно встретить рекомендации о целесообразности определения концентрации ТТГ и тиреоглобулина для оценки степени тяжести ЙДЗ. Концентрация ТТГ является индикатором для выявления неонатального гипотиреоза, но его эффективность как критерия ЙДЗ в старших возрастных группах спорна. Причиной повышения ТТГ могут явиться заболевания ЩЖ, прием ряда медикаментов и др. Кроме того, у взрослых из эндемичных районов уровни ТТГ могут быть более низкими, чем из йодобеспеченных, за счет формирования автономно функционирующей ткани ЩЖ.

В таблице 9 суммированы все эпидемиологические критерии, в той или иной степени используемые для оценки выраженности йодного дефицита.

**Table:** Таблица 9. Эпидемиологические критерии оценки степени тяжести йододефицитных заболеваний

Критерии	Отсутствие йододефицита	Степень тяжести йодного дефицита
легкая	средняя	тяжелая
% зоба у школьников (пальпация или УЗИ)	<5	5,0–19,9	20,0–29,9	>30,0
Медианная концентрация I в моче, мкг/л	>100	50–99	20–49	<20
Частота ТТГ>5 мЕ/л при неонатальном скрининге, %	<3	3,0–19,9	20,0–39,9	>40,0
Медиана тиреоглобулина у детей и взрослых, нг/мл	<10	10,0–19,9	20,0–39,9	>40,0

Для того чтобы судить об исходной тяжести ЙД, необходимо иметь как минимум два параметра.

В том случае, если в регионе уже проводятся мероприятия по йодной профилактике, для оценки их эффективности достаточно оценивать уровень экскреции йода с мочой и учитывать количество семей, использующих в питании йодированную соль.

 

## ПРИЛОЖЕНИЕ

**Table:** Критерии оценки качества медицинской помощи

1	Выполнено ультразвуковое исследование щитовидной железы	2	А
2	Выполнено исследование уровня тиреотропного гормона в крови	5	C
3	Выполнена тонкоигольная аспирационная биопсия узловых образований щитовидной железы при наличии показаний	2	B
4	Выполнено исследование уровня кальцитонина в крови при узловых образованиях щитовидной железы	2	A
5	Выполнена оценка пунктата узлового образования с использованием шести категорий классификации Бетесда	5	C
6	Выполнено МСКТ/МРТ шеи при подозрении на компрессионный синдром	5	C
7	Выполнена сцинтиграфия щитовидной железы при подозрении на функциональную автономию щитовидной железы	5	C
8	Выполнено назначение консервативной терапии при диффузном зобе при наличии показаний	5	С
9	Выполнено хирургическое лечение при наличии показаний	5	C
10	Выполнена терапия радиоактивным йодом при наличии показаний	1	A
